# When and How Ingratiation Boosts Coworker-Directed Cooperative Behavior

**DOI:** 10.3390/bs16060978

**Published:** 2026-06-12

**Authors:** Yun Chen, Min Cui

**Affiliations:** 1 College of Management, Ningbo University of Finance and Economics, Ningbo 315174, China; chenyun2@nbufe.edu.cn; 2 Business School, University of International Business and Economics, Beijing 100029, China

**Keywords:** ingratiation, moral rumination, cooperative behavior, moral identity, moral cleansing theory

## Abstract

Drawing on moral cleansing theory, this study adopts an actor-centered perspective to examine how ingratiation relates to employees’ moral rumination and subsequent coworker-directed cooperative behavior, thereby offering insights to help organizations to understand and guide such behaviors. Using a multi-wave survey design, this study collected data from 272 employees to examine a theoretical model investigating how employee ingratiation influences coworker-directed cooperative behavior through moral rumination, while also examining the moderating role of employee moral identity. The results indicate that employee ingratiation positively influences moral rumination, which in turn enhances coworker-directed cooperative behavior. Furthermore, the indirect effect of ingratiation on coworker-directed cooperative behavior via moral rumination is strengthened among employees with high moral identity. This study advances the literature by shifting the focus from targets and observers to actors themselves, examining how ingratiation shapes actors’ own moral perception and subsequent behavior. It further contributes by introducing moral rumination as a mediating mechanism and exploring the moderating effect of moral identity, as well as offering new insights into ingratiation in organizational contexts.

## 1. Introduction

Ingratiation, known as “strategic impression management behavior,” encompasses a set of tactics such as other-enhancement, opinion conformity, self-presentation, and rendering favors (Bande et al., 2019). Although ingratiation does not necessarily involve deception or insincerity, it is generally regarded as a strategic form of impression management aimed at influencing how one is perceived by others (Fletcher, 1992; Rosenfeld, 1997). Its core purpose lies not in genuine social interaction, but in enhancing the ingratiator’s appeal to resource-holding targets (such as supervisors) in order to secure resources, opportunities, and relational benefits (Jones & Pittman, 1982).

Ingratiation has a long tradition in impression management research, with studies examining its effects from actor–target perspectives (Li & Cui, 2026). Research indicates that upward ingratiation can enhance leader-member exchange (Kim et al., 2018), improve performance evaluations (Asadullah et al., 2023), strengthen in-group status (De Clercq et al., 2021; Yang et al., 2024), and facilitate salary growth, promotions, and career success (Bolino et al., 2016; Proost et al., 2010; Sibunruang et al., 2016; Wu et al., 2025). However, ingratiation may also trigger negative outcomes, such as superiors’ social undermining (Keeves et al., 2017) and counterproductive work behaviors (Yan et al., 2020), even hinder newcomer socialization when it is excessive (Wu et al., 2025).

Although research on the consequences of ingratiation is relatively well developed, less attention has been devoted to its potential moral implications for those who engage in it. Because ingratiation is often enacted with instrumental goals in mind (Cheng et al., 2024), it may prompt actors to reflect on how their behavior aligns with their personal values and moral standards. Existing research suggests that observers’ interpretations of ingratiation—whether viewed as authentic or tactical—shape evaluations of the ingratiator’s trustworthiness (Long, 2021). Likewise, third-party employees may sometimes perceive ingratiators as self-interested or manipulative when they view such behavior as threatening their own status or opportunities (Cheng et al., 2024). However, prior studies have focused primarily on these external evaluations while paying relatively little attention to how ingratiators themselves interpret and evaluate their actions.

Individuals who engage in ingratiation are often aware of the instrumental motives underlying their behavior. Such awareness may encourage self-reflection regarding the appropriateness and implications of their conduct, particularly because people generally seek to maintain a positive moral self-concept (Monin & Jordan, 2009) and to behave in ways that are consistent with their ethical standards (Mazar et al., 2008). Rather than assuming that ingratiation necessarily threatens one’s moral self-image, we suggest that engaging in ingratiation may stimulate moral reflection and cognitive evaluation of one’s behavior. Examining these self-reflective processes may therefore provide a useful perspective for understanding how ingratiation shapes subsequent workplace behavior toward coworkers who are not the direct targets of the ingratiation attempt.

To address these important research questions, we developed a theoretical model to examine how ingratiation elicits moral responses among actors. Drawing on moral cleansing theory, we propose that employees who actively engage in ingratiation may experience heightened moral rumination—a cognitively self-reflective state in which individuals question the moral implications of their behavior. This heightened awareness may subsequently be associated with greater coworker-directed cooperative behavior, a pattern that is theoretically consistent with the process described by moral cleansing theory.

When employees engage in ingratiation for instrumental purposes, they may become concerned that supervisors will recognize their underlying intentions and evaluate their behavior unfavorably (Fletcher, 1992; Rosenfeld, 1997), while also gradually realizing that their actions may disadvantage coworkers (Cheng et al., 2024; Li & Cui, 2026). The combined influence of these concerns may make it psychologically difficult for employees to disengage from thoughts about their behavior, leading them to experience moral rumination—a persistent tendency to question whether their actions are ethically appropriate, whether they may negatively affect others, and whether their true motives have been revealed (Brosschot et al., 2006; Yuan et al., 2018). Consequently, when ingratiators experience moral rumination, they are more likely to subsequently engage in cooperative behavior toward coworkers (Parks et al., 2003; Sachdeva et al., 2009).

**Figure 1:**
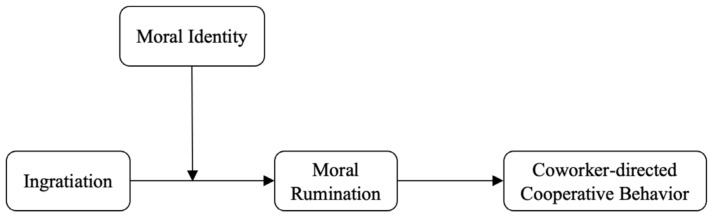
Theoretical Model.

Furthermore, moral cleansing theory suggests that individuals differ in their responses to their conduct (Martens et al., 2010). Building on this perspective, we argue that the influence of ingratiation on moral rumination and cooperative behavior may vary depending on individuals’ tendencies to perceive such behavior. Accordingly, we propose moral identity as a potential boundary condition for the effects of ingratiation. Employees with high moral identity place greater emphasis on ethical standards, tend to act in accordance with their moral principles, and exhibit heightened sensitivity to threats related to their moral image (Aquino et al., 2009; Aquino & Reed, 2002). High moral identity employees are more inclined to reflect on the motives behind their ingratiating behavior—considering whether they manipulated their supervisor’s impressions and adversely affected their colleagues’ interests—leading to increased moral rumination. This, in turn, may motivate them to engage in more cooperative behaviors as a way to address the cognitive tension arising from moral rumination. Therefore, we expect that moral identity may amplify the moral cognitive reactions triggered by ingratiation. Figure 1 presents our theoretical model.

This study contributes to the literature in several ways. First, existing research on ingratiation has primarily concentrated on its benefits for actors (Kim et al., 2018; Sharma & Dhar, 2022) and its influence on the moral evaluations of targets and observers (Cheng et al., 2024; Long, 2021), while the moral consequences for the actors themselves have been underexplored. This study examines the psychological and subsequent behavioral effects of ingratiation on the actors from an ethical perspective, thereby advancing current understanding of this behavior.

Second, we also contribute to moral cleansing theory by extending its applicability to social behaviors driven by instrumental motives. Although moral cleansing has traditionally been examined in contexts of explicit unethical conduct (Wang et al., 2022), we apply this framework to ingratiation—a self-interested behavior. We propose that moral rumination (cognitive reflection on the morality of one’s ingratiation) can serve as a precursor to subsequent cooperative behavior.

Finally, this study also contributes to the field of moral identity by examining its role in strengthening the moral rumination process. Specifically, the findings indicate that individuals with a strong moral identity are more inclined to reflect on their own ingratiating behavior, which triggers more intense moral rumination and subsequent corrective actions aimed at restoring their moral self-image (Sachdeva et al., 2009; Simbrunner & Schlegelmilch, 2017). By clarifying how moral identity facilitates the restoration of an individual’s moral self-image through targeted compensatory behaviors, this research enriches the literature on moral identity.

## 2. Theory and Hypothesis Development

### 2.1. Moral Cleansing Theory

Moral cleansing theory suggests that individuals hold internalized standards regarding what it means to be a moral person (Wang et al., 2022). According to the theory, people generally seek to maintain consistency between their behaviors and valued moral characteristics such as honesty, caring, and helpfulness (Aquino & Reed, 2002). Prior research has argued that when individuals perceive a discrepancy between their behavior and these standards, they may become more attentive to the moral implications of their actions and engage in various forms of self-regulation (West & Zhong, 2015; Zhong & Liljenquist, 2006). In this sense, moral cleansing theory provides a useful framework for understanding how people respond to morally relevant self-evaluations. Existing research further suggests that such responses may take different forms, including actions directed toward others and broader prosocial or altruistic behaviors (Sachdeva et al., 2009; Simbrunner & Schlegelmilch, 2017; West & Zhong, 2015). Overall, the theory highlights the importance of moral self-regulation and individuals’ efforts to maintain a positive moral self-concept.

Much of the existing literature has examined moral cleansing through affective mechanisms. For example, leaders who engage in abusive supervision may subsequently display more constructive leadership behaviors in response to feelings of guilt (Liao et al., 2018). Likewise, employees who engage in unethical pro-organizational behavior may exhibit increased voice behavior following experiences of moral unease (Wang et al., 2022). At the same time, emerging research suggests that morally relevant self-regulation may also involve cognitive processes. For instance, Pan et al. (2024) found that employees who engaged in knowledge hiding subsequently experienced changes in moral credit perceptions that were associated with later behavioral responses. These findings suggest that cognitive reflection may represent one pathway through which individuals evaluate and respond to the moral implications of their behavior.

Drawing on moral cleansing theory, we propose that ingratiation may stimulate moral rumination because employees are often aware of the instrumental motives underlying such behavior (Cheng et al., 2024). Engaging in ingratiation may therefore prompt employees to reflect on whether their actions align with their personal values and moral standards (Barkan et al., 2015). Moral rumination captures this self-reflective process, in which individuals consider the ethical implications of their behavior. Moral cleansing theory further suggests that such moral reflection may encourage behaviors that are more consistent with a positive moral self-concept (Sachdeva et al., 2009; Simbrunner & Schlegelmilch, 2017). Accordingly, employees who experience greater moral rumination following ingratiation may be more likely to engage in cooperative behavior toward coworkers.

### 2.2. Ingratiation and Moral Rumination

Moral rumination refers to individuals’ repetitive reflection on whether their actions may harm others’ interests or violate moral standards (Brosschot et al., 2006). Based on moral cleansing theory, we propose that employee ingratiation is positively related to moral rumination.

As elaborated by Bazerman et al. (1998), individuals typically possess a “want self” and a “should self.” The “want self” is driven by emotions, impulses, and short-term goals, whereas the “should self” is associated with rationality, deliberation, and long-term objectives. In the context of ingratiation, the “want self” seeks others’ favor and immediate benefits (Friedlander & Schwartz, 1985), while the “should self” aspires to act as an ethical individual (Ruedy et al., 2013).

Employees engage in ingratiation—such as praising supervisors or proactively offering assistance—to gain favor from their superiors (Jones & Pittman, 1982). However, existing research indicates that the actual outcomes of such behavior may diverge from expectations. On one hand, the effectiveness of ingratiation largely depends on its perceived authenticity. If detected as instrumentally motivated, supervisors may view it as insincere and manipulative and label the ingratiator as a “calculating flatterer” (Cheng et al., 2024; Long, 2021; Turnley et al., 2013). On the other hand, colleagues may interpret ingratiation as unfair competition for limited resources and future promotion opportunities (Cheng et al., 2024; Leary, 1995), leading to negative reactions such as ostracism (Azeem et al., 2023; Cheng et al., 2024).

Consequently, the psychological pressure arising from internal value conflicts (Tsang, 2016), supervisory skepticism, and peer negative reactions may collectively prompt individuals to reflect on whether their behavior aligns with their personal values and moral standards. Such a discrepancy may make it psychologically difficult for individuals to disengage from thoughts about their ingratiating behavior, potentially leading them to experience moral rumination (Yuan et al., 2018)—persistently questioning whether their actions are ethical, whether they harm others’ interests, and whether their true motives have been detected. Although our study does not directly measure moral self-image threat or restorative motivation, this pattern is consistent with the broader logic of moral cleansing theory. Thus, we propose:

**Hypothesis 1.** 
*Employee ingratiation is positively related to moral rumination*.

### 2.3. The Mediating Role of Moral Rumination

Based on moral cleansing theory, we further propose that moral rumination following ingratiation may be associated with increased cooperation toward colleagues who are not the targets of ingratiation. Specifically, we suggest that ingratiation is positively related to coworker-directed cooperative behavior via moral rumination. Coworker-directed cooperative behavior refers to actions and interactions that promote collaboration and support within the workplace (Chang et al., 2017).

More specifically, moral rumination involves employees reflecting on their own behavior and its moral implications. Such reflection may encourage individuals to consider the consistency between their actions and their personal moral standards. In turn, this reflective process may be associated with behaviors that are oriented toward supporting coworkers. Although our study does not directly measure restorative motivation or moral compensation, this pattern is theoretically consistent with a moral cleansing interpretation.

Cooperative behaviors—such as offering help to colleagues, sharing work-related information, and collaborating to solve work-related challenges (Chang et al., 2017; Sanders & Schyns, 2006)—can serve as prosocial actions that may help individuals restore a sense of moral balance, according to the logic of moral cleansing theory (Simpson & Willer, 2015; Castañer & Oliveira, 2020). Through collaboration with colleagues, employees may maintain positive interactions while also potentially addressing concerns about their own moral self-image.

In summary, ingratiation may raise actors’ moral rumination—a cognitive awareness that their behavior carries potential moral ambiguity. As a result, employees may subsequently engage in coworker-directed cooperative behavior. We emphasize that moral rumination reflects a cognitive self-regulatory process, and the observed pattern is consistent with the expectations of moral cleansing theory. Thus, we propose:

**Hypothesis 2.** 
*Employee ingratiation has a positive indirect relationship with coworker-directed cooperative behavior via moral rumination*.

### 2.4. The Moderating Role of Moral Identity

While we propose that employee ingratiation may trigger moral rumination and subsequently relate to coworker-directed cooperative behavior, the strength of this relationship may depend on employees’ moral identity. Moral identity refers to the degree to which moral traits are central to an individual’s self-concept (Aquino & Reed, 2002). We suggest that moral identity may serve as a moderator in the relationship between ingratiation and moral rumination.

Individuals with a strong moral identity view characteristics such as honesty, kindness, and helpfulness as important components of who they are (Aquino & Reed, 2002). As a result, they tend to pay greater attention to the moral implications of their actions and are more likely to evaluate whether their behavior aligns with their personal values and standards (Kingsford et al., 2018; Youniss & Yates, 1999). When these individuals engage in ingratiation, their heightened sensitivity to moral considerations may lead them to engage in greater moral reflection, thereby increasing moral rumination.

In contrast, individuals with low moral identity place less emphasis on moral traits when defining themselves. Consequently, they may be less attentive to the moral implications of ingratiation and less likely to engage in extensive self-reflection regarding such behavior. As a result, the positive relationship between ingratiation and moral rumination is likely to be weaker for employees with low moral identity.

Based on the above discussion, we propose the following hypotheses:

**Hypothesis 3.** 
*Employee moral identity moderates the positive relationship between ingratiation behavior and moral rumination such that the relationship is stronger when employee moral identity is higher rather than lower*.

Integrating Hypotheses 2 and 3, we propose the following:

**Hypothesis 4.** 
*Employee moral identity moderates the positive indirect relationship between ingratiation behavior and coworker-directed cooperative behavior via moral rumination such that the indirect relationship is stronger when moral identity is high versus low*.

## 3. Method

### 3.1. Sample and Procedures

This study used fully anonymized survey data with no experimental intervention, no sensitive information, and no commercial purpose. Data were collected through an EDP (Executive Development Program) at a university in China using a snowball sampling approach. Participants represented a range of occupational backgrounds, including marketing, sales, administration, finance, operations, and technical positions across multiple industries, such as service, finance, manufacturing, and marketing-related sectors. All participants were independently recruited and participated in the program as individual professionals, rather than being nested within intact work teams or organizational sampling frames. Participants voluntarily completed the survey as individual professionals rather than as members of formally sampled organizational units or intact work teams. All data have been securely stored and used solely for research purposes.

To enable matching across three-time waves, we assigned each participant a unique pseudonym (based on their WeChat name and the last four digits of their mobile phone number). After matching was completed, all identifiers were removed, and the final data was fully anonymized prior to analysis. Thus, the data are better described as pseudonymously collected and subsequently anonymized.

In accordance with Article 32(2) of the *Measures for Ethical Review of Life Sciences and Medical Research Involving Human Subjects* (National Health Commission of the People’s Republic of China, No. 4 [2023]) (National Health Commission of the People’s Republic of China et al., 2023), this study was determined to be exempt from ethical review by the relevant institutional review board at the authors’ institution. The exemption applies because the study involves fully anonymized data, no experimental intervention, no sensitive personal information, and no commercial interests.

All participants were fully informed of the research purpose, procedures, potential risks, and their rights before the survey and signed informed consent forms; participation was entirely voluntary, and participants were free to withdraw at any time without penalty.

To mitigate concerns related to common method bias arising from self-reported data, we adopted a multi-wave survey design with three separate time points (Ployhart & Vandenberg, 2010). Following prior research (Walumbwa et al., 2018), we set a one-week interval between time points to balance common method bias control against participant attrition. The research began in January 2024, and the questionnaires were distributed at three time points, each one week apart. At T1, participants reported ingratiation and moral identity, as well as demographic variables (300). At T2, participants reported moral rumination (291, response rate = 97.0%). At T3, participants reported cooperative behavior with coworkers (272, response rate = 90.7%). Questionnaires from different time points were matched using pseudonyms (WeChat names) and the last four digits of mobile phone numbers.

After removing invalid questionnaires based on the following criteria—(1) excessive repetitive responses: selecting the same response option for more than seven consecutive items; (2) abnormal response times: completion time below 60 s or above 30 min; (3) unmatchable responses: failure to complete all three waves—a final sample of 272 participants who completed all three waves was obtained (final effective response rate = 272/300 = 90.7%). Among these 272 participants, 58.5% were female, the average age was 35.78 years (SD = 8.53), the average years of education was 15.31 years (SD = 2.49), and the average organizational tenure was 6.64 years (SD = 6.99).

### 3.2. Measures

As Brislin (1980) suggested, a “translate-back” procedure was conducted in this study. Full item wording is available in Appendix A.

#### 3.2.1. Ingratiation Behavior

We used the four items scale from Bolino and Turnley (1999) to measure employee ingratiation. A sample item was: “I praise my supervisor at work for his/her achievements so that he/she thinks I am a good person.” (1 = never to 5 = always; α = 0.92).

#### 3.2.2. Moral Rumination

To capture participants’ moral rumination, we adapted a 3-item scale from Kundro (2023), adapted from Trapnell and Campbell (1999)’s general rumination scale. A sample item was: “In the past week, I ruminate about the morality of my ingratiation behaviors.” (1 = strongly disagree to 5 = strongly agree; α = 0.85).

#### 3.2.3. Coworker-Directed Cooperative Behavior

We utilized a 4-item scale from Chang et al. (2017) to rate coworker-directed cooperative behavior. Sample item was: “During the past week, I shared work reports and documents with my colleagues in my work.” (1 = never to 5 = always; α = 0.87).

#### 3.2.4. Moral Identity

First, participants were asked to read about nine characteristics: empathy, compassion, fairness, friendliness, generosity, helpfulness, hard work, sincerity, and kindness. Then, following this, they rated a 5-item scale, developed by Aquino and Reed (2002), to assess their moral identity. An example item was: “Feeling good about being a person with these characteristics.” (1 = strongly disagree to 5 = strongly agree; α = 0.90).

#### 3.2.5. Control Variables

This study controls for participants’ gender (0 = male, 1 = female), age, years of education, and organizational tenure (in years) in the analyses, because previous research suggested that demographic factors can influence cooperative behavior (Zakaria et al., 2020). The variables were self-reported by the participants.

## 4. Results

The results of the confirmatory factor analysis conducted using SPSS 26.0 are presented in Table 1, in which the theoretical model fits the model better (*χ*^2^ = 198.97, *df* = 98, *χ*^2^/*df* = 2.03, CFI = 0.965, TLI= 0.957, RMSEA = 0.062, SRMR = 0.035) than alternative models.

Table 2 reports mean, standard deviation and the correlation among the core variables in this study. Results showed that ingratiation was positively correlation with moral rumination (*r* = 0.14, *p* < 0.05), and moral rumination was positively correlation with cooperative behaviors (*r* = 0.20, *p* < 0.01), primarily supporting H1 and H2.

Table 3 shows the results of the linear regression analyses conducted using SPSS 26.0. Results showed that ingratiation was positively associated with moral rumination (*b* = 0.21, *p* < 0.05), supporting Hypothesis 1^1^.

Hypothesis 2 proposes that moral rumination mediates the relationship between ingratiation and cooperative behavior. As shown in Table 3, moral rumination was positively related to cooperative behaviors (*b* = 0.17, *p* < 0.01). We further used the RMediation (https://amplab.shinyapps.io/MEDCI/?winzoom=1 (accessed on 13 June 2025)) method proposed by Tofighi and MacKinnon (2016) to test the mediating effect of moral rumination, and the result was significant (*b* = 0.04, 95% CI [0.003, 0.082]), supporting Hypothesis 2^2^. However, the direct effect of ingratiation on coworker-directed cooperative behavior remained substantial after including moral rumination (*b* = 0.57, *p* < 0.001). The estimated total effect was approximately 0.61, suggesting that the indirect pathway accounted for only a modest proportion of the overall relationship (approximately 6%). These findings indicate that moral rumination represents a partial, rather than dominant, explanatory mechanism linking ingratiation to coworker-directed cooperative behavior.

**Figure 2:**
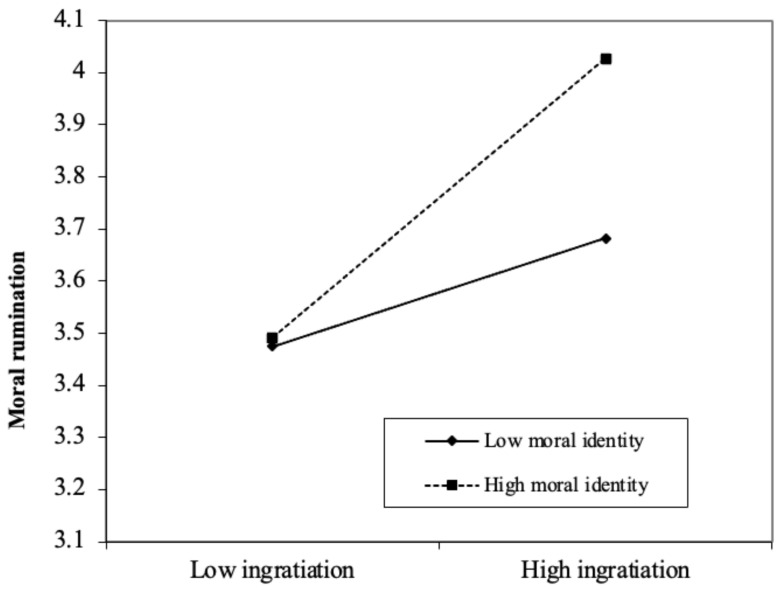
Interaction of Ingratiation and Moral Identity on Moral Rumination.

Hypothesis 3 proposes that moral identity moderates the relationship between ingratiation and moral rumination. In the formal analysis, the centered variables of ingratiation and moral identity were entered into the regression model. As shown in Table 3, there was a significant and positive interaction between ingratiation and moral identity on moral rumination (*b* = 0.29, *p* < 0.05)^3^. To further explore the moderating effect of moral identity, we plotted simple slopes. As Figure 2 shows, the positive effect of ingratiation on moral rumination was stronger when moral identity was high (+1SD; *b* = 0.47, *t* = 2.21, *p* < 0.05), whereas it was not significant when moral identity was low (*b* = 0.18, *t* = 1.82, *ns*), supporting Hypothesis 3.

Using SPSS 26.0, we further tested the conditional indirect effects. As shown in Table 4, results further indicated that the indirect effect of ingratiation on cooperative behavior via moral rumination was significant at high moral identity (*b* = 0.04, 95% CI [0.002, 0.084], not including 0), but not at low moral identity (*b* = −0.0004, 95% CI [−0.042, 0.026]). contains 0) and the difference between them is significant (*b* = 0.02, 95% CI [0.001, 0.081] does not contain 0), supporting Hypothesis 4 4.

## 5. Discussion

Drawing on moral cleansing theory, this study explored when and how employee ingratiation promotes coworker-directed cooperative behavior. A three-wave field study of 272 employees found that ingratiation is positively related to moral rumination, which in turn promotes coworker-directed cooperative behavior. Additionally, moral identity moderates this indirect effect: the positive relationship between employee ingratiation and coworker-directed cooperative behavior via moral rumination is significant only when moral identity is high.

### 5.1. Theoretical Implications

First, adopting an actor-centric perspective, this study suggests that ingratiation is associated with subsequent coworker-directed cooperative behavior through moral rumination. Prior research has long focused on reputation-related outcomes (e.g., others’ evaluations and social likability; Cheng et al., 2024; Kim et al., 2018; Long, 2021; Sharma & Dhar, 2022) while overlooking the moral processes within the actors themself. Echoing Harvey’s (2025) work on self-promotion, we draw on moral cleansing theory to propose a process whereby ingratiation relates to increased moral rumination, which in turn is associated with greater coworker-directed cooperative behavior. This pattern is theoretically consistent with a moral cleansing interpretation and deepens our understanding of how ingratiation may relate to actors’ own moral attitudes and behaviors.

Second, we extend moral cleansing theory to instrumentally motivated social behaviors. While prior research has primarily examined explicit unethical acts (e.g., Pan et al., 2024; Wang et al., 2022; Zhong & Liljenquist, 2006), our findings indicate that even self-interested behaviors such as ingratiation can be associated with moral rumination and subsequent cooperative behavior toward coworkers. Although our study does not directly measure psychological restoration, moral compensation, or moral realignment, this pattern is consistent with the broader logic of moral cleansing theory. This advances our understanding that ingratiators may reflect on the strategic nature underlying their ingratiation and respond with cooperative behavior.

Finally, we identify moral identity as a key boundary condition in the relationship between ingratiation and moral rumination. Although prior research has examined moderators such as political skill that can enhance the positive effects of ingratiation (Xu et al., 2023), few studies have investigated individual differences in ingratiators’ responses from a moral perspective. By testing the moderating role of moral identity, we reveal that the indirect effect of ingratiation on coworker-directed cooperative behavior via moral rumination is stronger among employees with high moral identity. These findings deepen the understanding of boundary conditions and respond to the relevant call from scholars (Jennings et al., 2015).

### 5.2. Managerial Implications

The research provides valuable insights for organizations. First, we find that ingratiation can, in certain contexts, facilitate cooperative behavior. However, this does not imply encouraging or endorsing ingratiation. Instead, managers should reduce employees’ need for strategic impression management—for example, by establishing transparent and fair performance evaluation and promotion systems to lower the incentive for employees to seek resources through ingratiation. At the same time, organizations should encourage ethical reflection and authentic cooperation, guiding employees toward positive interactions based on genuine contributions through training and role modeling.

Second, this study finds that moral identity amplifies the positive effects of ingratiation. Managers can cultivate employees’ moral identity to promote these beneficial aspects. For instance, aligning organizational reward, punishment, and promotion policies with moral standards encourages adherence to ethical norms and strengthens moral identity. Additionally, managers should foster a positive moral culture through training and communication, promoting moral discourse and collective commitment to ethical standards. Managers’ own ethical behavior and words will further inspire employees and reinforce their moral identity.

### 5.3. Limitations and Future Research

This research inevitably has several limitations. First, although cooperative behavior may be influenced by social desirability bias, self-report measures are commonly used to assess such workplace behaviors. However, self-report methods may raise concerns about common method bias (Podsakoff et al., 2012). To mitigate this bias, we adopted a multi-wave design with time intervals. Even so, self-report data still have inherent limitations for causal inference (Antonakis et al., 2010). In addition, self-reported cooperation may not accurately reflect employees’ actual helping behavior toward coworkers. Future research could use coworker or supervisor ratings as more robust measures, or introduce experimental designs to test our model and provide causal evidence.

Second, although this study tests the mediating role of employee moral rumination, other potential mechanisms may still exist. This study did not directly measure emotional components such as guilt or ethical dissonance. Moral rumination primarily reflects cognitive moral reflection rather than fully representing moral repair motivation. Future research should include measures of both cognitive and emotional components of moral cleansing. In addition, negative psychological states that may be triggered by ingratiation, such as guilt (Perkins et al., 2024), as well as affective or emotional dimensions, are also worthwhile avenues for exploration.

Third, we tested the moderating effect of moral identity. However, factors at the team level, such as competitive climate or Leader-Member Exchange (LMX) differentiation, may also influence employees’ perceptions of ingratiation (Cheng et al., 2024). For instance, a strong team competition climate may prompt employees to rationalize ingratiation, thereby reducing moral considerations. Future research is recommended to further investigate other moderators.

Finally, acknowledging the plausibility of an alternative explanation suggested by the positive correlation between ingratiation and moral identity—namely, that individuals with stronger moral awareness may be more likely to engage in ingratiation—future research may benefit from employing longitudinal designs to more carefully examine whether moral identity also serves as an antecedent of ingratiation and to assess the possibility of reciprocal relationships between these constructs.

## Figures and Tables

**Table 1:** Confirmatory Factor Analysis.

Models	*χ* ^2^	*df*	*χ*^2^/*df*	CFI	TLI	RMSEA	SRMR
Ingratiation, Moral Rumination, Cooperative Behavior, Moral Identity	198.97	98	2.03	0.965	0.957	0.062	0.035
Ingratiation + Moral Rumination, Cooperative Behavior, Moral Identity	572.20	101	5.67	0.837	0.806	0.131	0.109
Ingratiation + Moral Rumination, Cooperative Behavior + Moral Identity	1243.64	103	12.07	0.606	0.541	0.202	0.169
Ingratiation + Moral Rumination + Cooperative Behavior + Moral Identity	2064.55	104	19.85	0.322	0.218	0.263	0.218

*Note*. *N* = 272. “+” indicates that the variables are loaded on one single factor.

**Table 2:** Mean, Standard Deviation and the Correlation among the Core Variables.

	*Mean*	*SD*	1	2	3	4	5	6	7
1. Gender	0.58	0.49							
2. Age	35.78	8.52	−0.33 **						
3. Education	15.31	2.49	0.13 *	−0.24 **					
4. Job tenure	6.64	6.99	−0.21 **	0.61 **	−0.13 *				
5. Ingratiation	3.88	0.57	−0.13 *	0.05	−0.02	0.03			
6. Moral Rumination	3.51	0.89	−0.03	0.04	−0.03	0.02	0.14 *		
7. Cooperative Behavior	3.71	0.77	−0.09	0.04	0.09	0.002	0.62 **	0.20 **	
8. Moral Identity	4.26	0.64	−0.03	0.17 **	−0.09	0.07	0.24 **	0.14 *	0.26 **

*Note*. *N* = 272. For gender, male = 0, female = 1. * *p* < 0.05, ** *p* < 0.01.

**Table 3:** Regression Analysis.

Variable	Moral Rumination			Cooperative Behavior	
	Model 1	Model 2	Model 3	Model 4	Model 5
Gender	−0.03 (0.12)	−0.002 (0.12)	−0.01 (0.12)	−0.15 (0.10)	−0.14 (0.10)
Age	0.004 (0.01)	0.004 (0.01)	0.001 (0.01)	0.01 (0.01)	0.01 (0.01)
Education	−0.01 (0.02)	−0.01 (0.02)	−0.01 (0.02)	0.03 (0.02)	0.04 (0.02)
Tenure	−0.001 (0.01)	−0.001 (0.01)	−0.001 (0.01)	−0.01 (0.01)	−0.004 (0.01)
Ingratiation		0.21 * (0.10)	0.18 0.10)		0.57 *** (09)
Moral Identity			0.18 * (0.09)		
Ingratiation × Moral Identity			0.29 * (0.14)		
Moral Rumination					0.17 ** (0.05)
Intercept	3.53 *** (0.50)	2.69 *** (0.62)	3.58 *** (0.67)	3.11 *** (0.43)	2.51 *** (0.46)
*R* ^2^	0.003	0.02 *	0.05	0.02	0.06
*Adjusted R* ^2^	−0.01	0.002	0.02	0.01	0.04
*F*	0.17	1.13	1.81	1.43	3.34 **

*Note*. *N* = 272. For gender, male = 0, female = 1. * *p* < 0.05, ** *p* < 0.01, *** *p* < 0.001. The values presented in the table are unstandardized regression coefficients, with standard errors in parentheses.

**Table 4:** Moderated mediation effect analysis.

Moderator Variable	Indirect Effect	95% Confidence Interval
	Ingratiation → Moral Rumination → Cooperative Behavior	
High moral identity	0.04	[0.002, 0.084]
Low moral identity	−0.0004	[−0.042, 0.026]
Index	0.02	[0.001, 0.081]

*Note*: M ± 1SD indicates mean plus or minus 1 standard deviation, Bootstrap sample size is 5000.

## Data Availability

The data presented in this study are available on request from the corresponding author due to privacy and ethical considerations.

## Notes

1 We also evaluated the relationship between the primary variables without control variables (see Becker, 2005), and all hypotheses remained supported. Ingratiation was significantly related to moral rumination (*b* = 0.22, *p* < 0.05). Moral rumination was significantly related to cooperative behavior (*b* = 0.10, *p* < 0.05).

2 We removed control variables and evaluated the indirect effect of ingratiation on cooperative behavior via moral rumination was significant (estimate = 0.02, 95% CI = [0.001, 0.052]).

3 The interaction term between ingratiation and moral identity in predicting moral rumination (*b* = 0.29, *p* < 0.05) remained significant.

4 We further removed control variables and evaluated the indirect effect of ingratiation on cooperative behavior via moral rumination was significant at high moral identity (*b* = 0.04, 95% CI [0.002, 0.084], not including 0), but not at low moral identity (*b* = −0.0004, 95% CI [−0.042, 0.026]). contains 0) and the difference between them is significant (*b* = 0.02, 95% CI [0.001, 0.081] does not contain 0).

## Appendix A. All Measurement Items

**T1: Ingratiation toward supervisor (Bolino & Turnley, 1999)**

To my supervisor,

1. I praise my supervisor at work for his/her achievements so that he/she thinks I am a good person.
2. I do personal favors for my supervisor at work to show that I am friendly.
3. I compliment my supervisor at work to make him/her think I am likable.
4. I take an interest in my supervisor’s personal life to show that I am friendly.

**T1: Moral Identity (Aquino & Reed, 2002)**

This question requires your imagination. First, we have selected nine words to describe a person. This person could be you or someone else. Please read the following nine words: **compassionate, kind, fair, friendly, generous, helpful, hardworking, sincere, warm-hearted.** Then, imagine in your mind a person who possesses these characteristics. Imagine how such a person would think, feel, and act. Once you have a clear image of this person, please rate your agreement with the following statements:

1. Being a person who has these characteristics would make me feel good.
2. These characteristics are an important part of who I am.
3. I would feel proud to have these characteristics.
4. Having these characteristics is very important to me.
5. I strongly desire to have these characteristics.

**T2:** Moral Rumination **(adapted from Kundro, 2023)**

In the past week,

1. I ruminate about the morality of my ingratiation behaviors.
2. I spend a great amount of time thinking about whether my ingratiation behavior at work is moral or not.
3. I ruminate or dwell on whether I did the right or wrong thing when engaging in ingratiation at work.

**T3:** Coworker-Directed Cooperative Behavior **(Chang et al., 2017)**

During the past week,

1. I share work reports and documents with my colleagues in my daily work.
2. I share work experiences or tips with my colleagues in my daily work.
3. I keep my colleagues informed of news that I know in my daily work.
4. I share my professional skills with my colleagues in my daily work.
